# Classification of Activities of Daily Living Based on Grasp Dynamics Obtained from a Leap Motion Controller

**DOI:** 10.3390/s22218273

**Published:** 2022-10-28

**Authors:** Hajar Sharif, Ahmadreza Eslaminia, Pramod Chembrammel, Thenkurussi Kesavadas

**Affiliations:** Department of Mechanical Science and Engineering, Health Care Engineering Systems Center, The Grainger College of Engineering, University of Illinois at Urbana-Champaign, Champaign, IL 61820, USA

**Keywords:** Leap Motion Controller, activities of daily living, hand grasps classification

## Abstract

Stroke is one of the leading causes of mortality and disability worldwide. Several evaluation methods have been used to assess the effects of stroke on the performance of activities of daily living (ADL). However, these methods are qualitative. A first step toward developing a quantitative evaluation method is to classify different ADL tasks based on the hand grasp. In this paper, a dataset is presented that includes data collected by a leap motion controller on the hand grasps of healthy adults performing eight common ADL tasks. Then, a set of features with time and frequency domains is combined with two well-known classifiers, i.e., the support vector machine and convolutional neural network, to classify the tasks, and a classification accuracy of over 99% is achieved.

## 1. Introduction

Many neurological conditions lead to motor impairment of the upper extremities, including muscle weakness, altered muscle tone, joint laxity, and impaired motor control [1,2]. As a result, common activities such as reaching, picking up objects, and holding onto them are compromised. Such patients will experience disabilities when performing activities of daily living (ADLs) such as eating, writing, performing housework, and so on [2].

Several evaluation methods are commonly being used to assess problems in performing ADLs [3,4,5]. Despite the wide application of these methods, all of them are subjective techniques, i.e., they are either questionnaires or qualitative scores assigned by a medical professional [3,5]. We hypothesize that providing a more quantitative metric could enhance the evaluation of the rehabilitation progress and lead to a more efficient rehabilitation regimen tailored to the specific needs of each individual patient.

**Figure 1:**
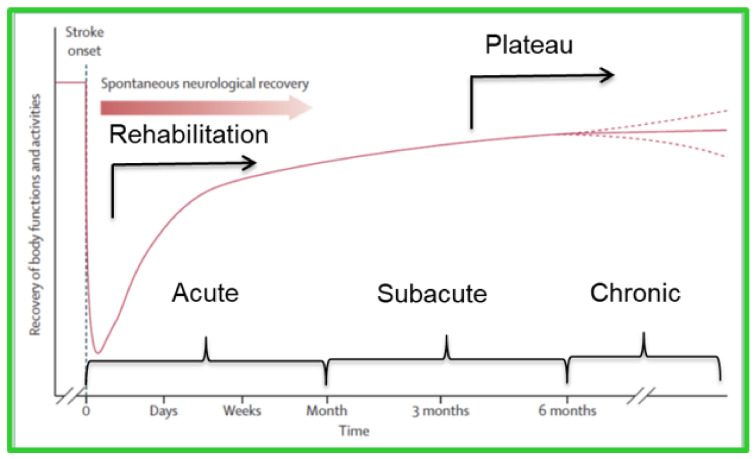
Stroke timeline [3].

For instance, a quantitative methodology could help to defer the plateau in the patient’s recovery. ‘Plateau’ is a term that is used to explain a stage of stroke recovery at which functional improvement is not observed (see Figure 1) and is determined by clinical observations, empirical research, and patient reports. In spite of the importance of plateau time as an indication of the time to discharge a patient from post-stroke physiotherapy, researchers have questioned the reliability of current methods for determining the plateau [6,7]. Demain et al. [6] implemented a standard critical appraisal methodology and found that the definition of recovery is ambiguous. For instance, there is a 12.5–26 week variability in plateau time for ADLs. A few parameters have been attributed with causing such inconsistency, among which, the qualitative nature of the assessment metrics can be mentioned [6,7,8]. An early and unnecessary discharge from physiotherapy can leave the patient with a permanent, yet potentially preventable, disability and having a more reliable technique to indicate the start of the plateau could help to determine the time at which to adjust the rehabilitation regimen and minimize neuromuscular adaptations which, in turn, can delay the plateau [8].

The term “Activities of Daily Living” has been used in many fields, such as rehabilitation, occupational therapy, and gerontology, to describe a patient’s ability to perform daily tasks that allow them to maintain unassisted living [9]. Since this term is very qualitative, researchers have proposed many subcategories of ADL, such as physical self-maintenance, activities of daily living, and instrumental activities of daily living [10], to assist physicians or occupational therapists in evaluating the patient’s ability to perform ADLs in a more justifiable fashion [9,11,12].

A fundamental step towards developing a quantitative ADL assessment methodology is to distinguish different ADL tasks based on hand gesture data. Based on the hardware applied to detect hand gestures, hand gesture recognition (HGR) methods can be divided into sensor-based and vision-based categories [13]. In sensor-based methods, the equipment used for data collection is exposed to the user’s body, whereas in vision-based techniques, different types of cameras are used for data acquisition [14,15]. Vision-based methods do not interfere with the natural way of forming hand gestures; however, several factors such as the number and positioning of cameras, the hand visibility, and algorithms applied on the captured videos can affect the performance of these techniques [13].

The Leap Motion Controller (LMC) is a marker-free vision-based hand-tracking sensor that has been shown to be a promising tool for HGR applications [16,17]. Several researchers have used the LMC to detect signs using hand gestures for American [18,19], Arabic [20,21,22,23], Indian [24,25,26], and other sign languages [27,28,29,30,31,32]. LMC has applications in education [33] and navigating robotic arms [34,35]. Researchers have investigated LMC applications in medical fields [36,37] including, but not limited to, upper extremity rehabilitation [38,39,40,41], wheelchair maneuvering [42], and surgery [43,44]. Bachmann et al. [45] reviewed the application of LMC for a 3D human–computer interface, and some studies have focused on the use of LMC for real-time HGR [46,47].

In this study, we used data collected from healthy subjects to develop the first stage of quantitative techniques that have a wide range of applications in improving the outcomes of assessments of many common neurological conditions. We demonstrated two classification schemes based on SVM and CNN that can efficiently classify ADL tasks. These classifiers use the features extracted by existing feature engineering methods from the collected data. In addition, we generated a dataset containing hand motion data collected using LMC while the participants performed a variety of common ADL tasks. We tested the performance of the proposed classification schemes using this dataset.

The tasks selected from this dataset included a variety of ADLs associated with physical self-maintenance, e.g., utilizing a spoon, fork, and knife, and activities of daily living, e.g., writing. In addition, based on Cutkosky grasp taxonomy, the tasks in this study include precision grasps, such as holding a pen, spoon, and spherical doorknob as well as power grasps like holding glass, a knife, and nail clippers [5,48,49]. These tasks involve diverse palm/finger involvement and facilitate the analysis of hand grasp over the entire range of motion that is typically used in ADLs.

## 2. Materials and Methods

### 2.1. Subjects and Data Acquisition

**Figure 2:**
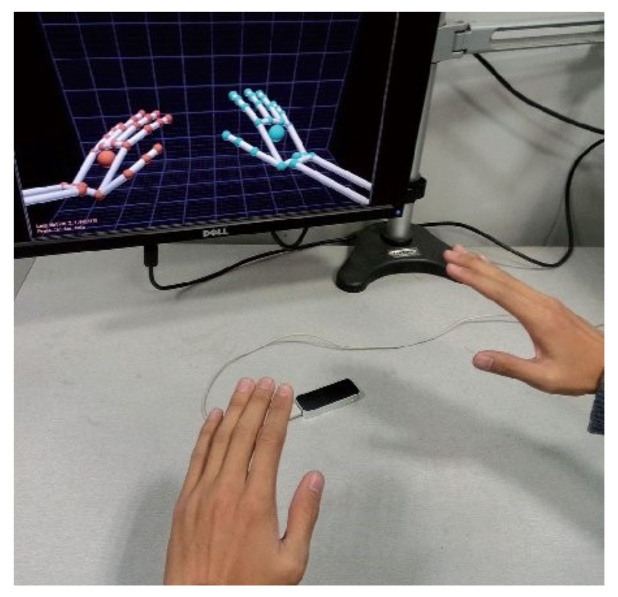
Leap Motion Controller connected to a computer that runs the Leap Motion Visualizer software showing the hands on top of the LMC camera [51].

**Figure 3:**
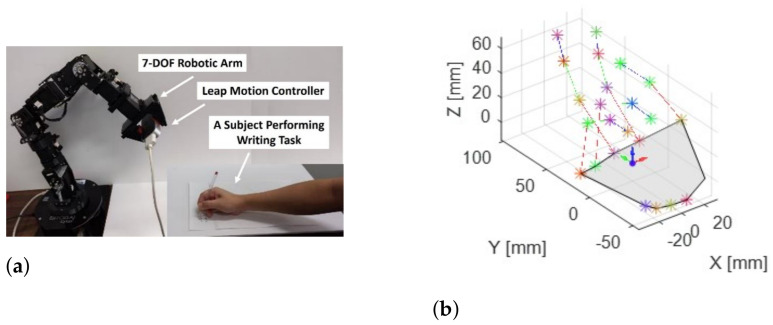
Experimental setup (**a**) and hand model in the global coordinate system (**b**).

**Figure 4:**
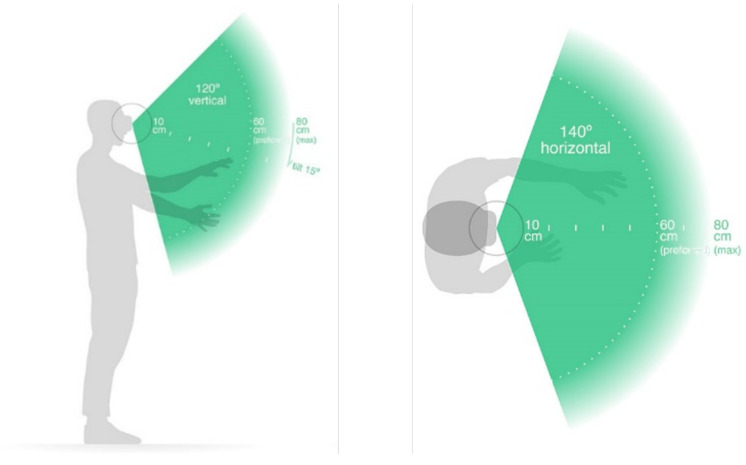
LMC’s interaction zone [53].

In this study, an LMC was employed to collect data from the dominant arm of the participants while they performed tasks. The LMC is a low-cost, marker-free, visual-based sensor that works based on the time-of-flight (TOF) concept for hand motion tracking. It contains a pair of stereo infrared cameras and three infrared LEDs. Using the infrared light data, the device creates a grayscale stereo image of the hands. As shown in Figure 2, the LMC is designed to either be placed on a surface, e.g., on an office desk, facing upward or be mounted on a virtual reality headset. To collect the ADL data, a 7-degrees-of-freedom robotic arm, i.e., Cyton Gamma 300 [50], was used to hold the LMC at an optimum position to minimize occlusion. The experimental setup and hand model in the LMC with the global coordinate system (GCS) are provided in Figure 3a,b, respectively. The LMC reads the sensor data and performs any necessary resolution adjustments in its local memory. Then, it streams the data to Ultraleap’s hand tracking software on the computer via a USB. It is compatible with both USB 2.0 and USB 3.0 connections. LMC’s interaction zone is between 10 cm and 80 cm from the device and has a 140° × 120° typical field of view, as shown in Figure 4 [32,51,52].

Nine healthy adults with intact hands, including three females and six males, were recruited to participate in this study, and informed consent was obtained from all participants. The age range of the participants was 25–62 years with an average of 37 years. This study was approved by the Institutional Review Board office of University of Illinois at Urbana-Champaign, and there were no limitations in terms of occupation, gender, or ethnicity when recruiting the participants.

**Figure 5:**
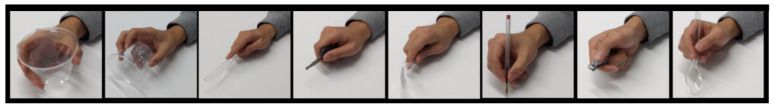
ADL tasks [54].

Each subject attended one session of data collection, and six of the participants completed two sets of tasks while two of them only completed one due to time limitations. Each set of tasks contained eight randomly distributed tasks, and the order of the tasks in the two sets was different. The subjects were asked to rest for 45 s between tasks to avoid muscle fatigue. During each task, the subjects were seated on a regular office chair with back support. Each task was performed with the participant’s dominant hand and was composed of static and dynamic phases. In the static phase, the participants were instructed to rest their forearms on a regular office desk to avoid tremor and hold an object, as listed in Table 1, for around 10 s, similar to how they would hold it in daily life. In the dynamic phase of the task, they were instructed to utilize the object over the entire range of motion that is usually performed in daily living at their own pace. Each dynamic task was repeated continuously 5 times without any rest intervals. Table 1 and Figure 5 demonstrate the ADL tasks.

### 2.2. Preprocessing

**Figure 6:**
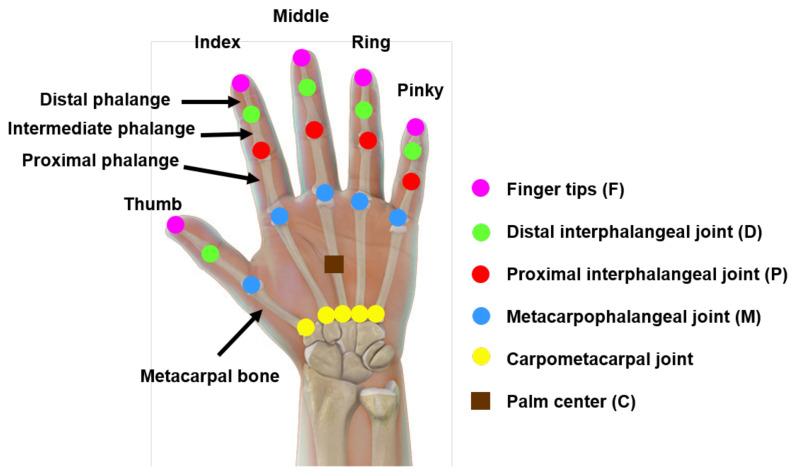
Hand joints and palm center [55].

**Figure 7:**
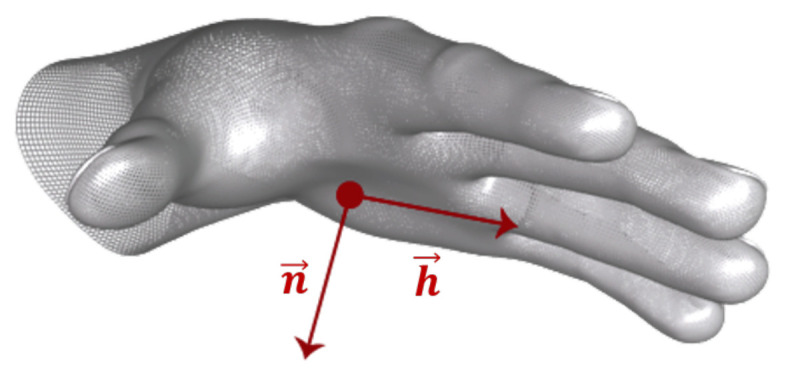
Hand coordinate system [56].

**Figure 8:**
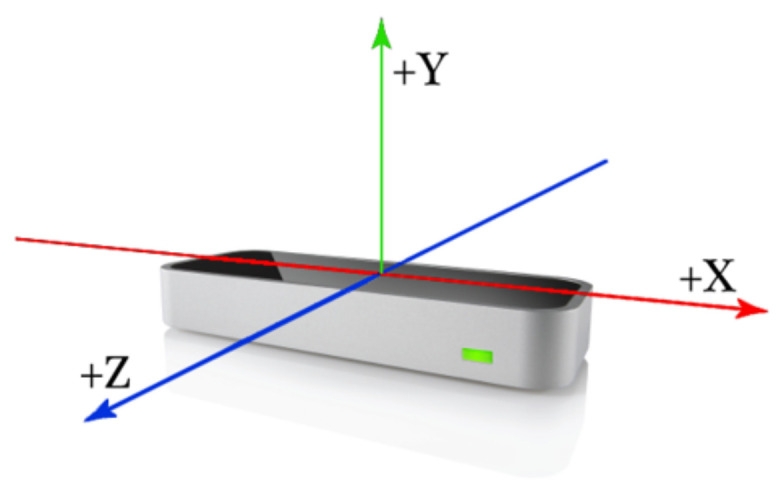
Leap Motion Controller frame of reference [57].

The LMC provides the coordinates of hand joints and the palm center, as demonstrated in Figure 6, in 3-dimensional space. It also provides the coordinates of three orthonormal vectors at the palm center, which form the hand coordinate system (HCS), as shown in Figure 7. These coordinates are in units of millimeters with respect to the LMC frame of reference. The origin of the LMC’s frame of reference is located at the top center position of the hardware, as presented in Figure 8. Therefore, while a participant performed a particular task, referred to as a trial hereafter, in each sample, i.e., each frame of the depth sensor, 84 coordinate values were recorded. The output of the LMC for each trial is a matrix of *n* × 84, where *n* is the number of samples, i.e., the number of frames.

#### 2.2.1. Change of Basis

The first preprocessing step was to transform LMC data from the LMC coordinate system to GCS using the Denavit–Hartenberg parameters [58] of the Cyton robot, since the LMC was rigidly attached to the end-effector of Cyton.

Once the LMC data had been transformed to GCS, the data were linearly translated into the hand palm center. Afterwards, by using a change of basis matrix at each frame, data were transferred from GCS to HCS based on Equation (1). In this equation, *A* is the change-of-basis matrix, or transition matrix, and its columns are the coordinates of the basis vectors of HCS in the GCS at each frame [59]. XHCS and XGCS are the data matrices in HCS and GCS, respectively.

(1) XHCS = inverse(A)∗XGCS

During the trials, the hand grasps, i.e., the relative positions and orientations of the fingers and palm, did not change. In this work, the hand grasps were used for classifying different ADL tasks. Therefore, upper limb trajectories during the dynamic phase of the tasks, e.g., the entire-hand motions from plate to mouth while performing the “spoon” task, captured in the GCS needed to be removed. Transforming data from GCS to HCS eliminated gross hand motions and left the hand grasp information.

#### 2.2.2. Filtering

At the next step, the transformed data were filtered using a median filter on a window size of 5 sampling points, i.e., 1/6 s.

### 2.3. Features and Classifiers

#### 2.3.1. Feature Extraction

The choice of features used to represent the raw data can significantly affect the performance of the classification algorithms [60]. In this work, three groups of features, as presented in Table 2, were calculated for each trial and later combined for classification. The features are explained in detail in the following text.

**Geometrical features in the time domain**

In order to compensate for different hand sizes, the features needed to be normalized. The geometrical features representing angles were divided by π, whereas the distance features were normalized to *M*. *M* is the accumulative Euclidean distance between the palm center and tip of the middle finger. At each sampling point, *M* was calculated by summation over the distance between the palm center and the metacarpophalangeal joint and the lengths of all three bones of the middle finger, as presented in Equation (2). Since there was less variation between participants’ hand grasps while performing the “cup” task, the coordinates of this task were used for the *M* calculation. The final length used for normalization was calculated by averaging *M* over the first 30 sampling points, i.e., the first second, of the first trial of the “cup” task.

(2) $$M\;=\;\left|\vec{CM}\right|+\left|\vec{MP}\right|+\left|\vec{PD}\right|+\left|\vec{DF}\right|$$

1. Adjacent Fingertips Angle (*AFA*): This feature demonstrates the angle between every two adjacent fingertip vectors, which is the angle between the vectors from the palm center to the fingertips. The *AFA* is calculated by Equation (3), where $F_{i}$ represents the fingertip location. This feature was normalized to the interval of [0, 1] by dividing the angles by π. Lu et al. [61] achieved a classification accuracy of 74.9% using the combination of this feature and the hidden conditional neural field (HCNF) as the classifier.
  (3) $$AFA\;=\;\;\frac{∠\left(F_{i},\;F_{i+1}\right)}{\pi }\;\;\;\;\;i\;=\;1,\;2,\ldots ,\;4\;$$
2. Adjacent Tips Distance (*ATD*): This feature represents the Euclidean distance between every two adjacent fingertips and is calculated by Equation (4), in which $F_{i}$ represents the fingertip location. There are four spaces between the five fingers of each hand, so there are four ATDs in each hand. This feature was normalized to the interval of [0, 1] by dividing the calculated distances by *M*. Lu et al. [61] achieved an accuracy level of 74.9% by using the combination of this feature and HCNF.
  (4) $$ATD\;=\;\frac{\left|F_{i}-F_{i+1}\right|}{M}\;\;\;\;\;i\;=\;1,\;2,\ldots ,\;4\;\;$$
3. Distal Phalanges Unit Vectors (DPUV) [62]: For each finger, the distal phalanges vector is defined as the vector from the distal interphalangeal joint to the fingertip, as presented in Figure 6. This feature was normalized by dividing by its norm.
4. Normalized Palm-Tip Distance (*NPTD*): This feature represents the Euclidean distance between the Palm Center and each fingertip. The *NPTD* is calculated by Equation (5) where $F_{i}$ represents the fingertip location, and C is the location of the palm center. This feature was normalized to the interval [0, 1] by dividing the distance by *M*. Lu et al. [61] achieved an accuracy level of 81.9% using the combination of this feature and HCNF, while Marin et al. [63] achieved an accuracy level of 76.1% using the combination of the Support Vector Machine (SVM) with the Radial Basis Function (RBF) kernel and Random Forest (RF) algorithms.
  (5) $$NPTD\;=\;\frac{\left|F_{i}-C\right|}{M}\;\;\;i\;=\;1,\;2,\ldots ,\;5\;\;\;\;$$
5. Joint Angle (JA) [64,65]: This feature represents the angle between every two adjacent bones at the interphalangeal and metacarpophalangeal joints. For example, for the distal interphalangeal joint, θ is derived by Equation (6).
  (6) $$\theta \;=\;\arccos\left(\frac{\vec{DF}\;.\;\;\vec{PD}}{\left|\vec{DF}\right|\left|\vec{PD}\right|}\right)\;$$
6. Fingertip-$\vec{h}$ Angle (*FHA*): This feature determines the angle between the vector from the palm center to the projection of every fingertip on the palm plane and $\vec{h}$, which is the finger direction of the hand coordinate system, as presented in Figure 8. *FHA* is calculated by Equation (7), in which $F_{i}^{p}$ is the projection of the $F_{i}$ on the palm plane. The palm plane is a plane that is orthogonal to the vector $\vec{n}$ and contains $\vec{h}$. By dividing the angles by π, this feature was normalized to the interval of [0, 1]. Lu et al. [61] and Marin et al. [63] achieved accuracy levels of 80.3% and 74.2% when classifying *FHA* features by HCNF and by using the combination of RBF-SVM with RF.
  (7) $$FHA\;=\;\;\frac{∠\left(F_{i}^{p}-C\;,\;h\right)}{\pi }\;\;\;\;i\;=\;1,2,\ldots ,5\;\;\;\;\;\;$$
7. Fingertip Elevation (*FTE*): Another geometrical feature is the fingertip elevation, which defines the fingertip distance from the palm plane. The *FTE* is calculated by Equation (8) in which “sgn” is the sign function, and $\vec{n}$ is the normal vector to the palm plane. Like previous features, the $F_{i}^{p}$ is the projection of the $F_{i}$ on the palm plane. Lu et al. [61] achieved an accuracy level of 78.7% using the combination of this feature and HCNF, while Marin et al. [63] achieved an accuracy level of 73.1% when classifying FTE features by the combination of SVM with the RBF kernel and RF.
  (8) $$FTE\;=\;\frac{sgn((F_{i}-F_{i}^{p}).\vec{n})∥F_{i}-F_{i}^{p}∥}{M}\;\;\;\;i\;=\;1,2,\ldots ,5\;\;\;\;\;\;$$

**Non-geometrical features in the time domain**

In order to compensate for the variations imposed by different participants’ hand sizes, the filtered data were normalized to *M*, which is described in the “geometrical features in the time domain” section. All non-geometrical time-domain features were calculated over a sliding window with a size of 15 samples, which equals 0.5 s, with no overlap between the windows.

1. Mean Absolute Value (*MAV*): The *MAV* was calculated by taking an average of the absolute values of the signal’s amplitude, using Equation (9). The MAV has shown promising results for classifying hand gestures [54,60,66,67].
  (9) $$MAV\;=\;\;\frac{1}{N}\sum_{n\;=\;1}^{N}{|X}_{n}|\;\;$$
2. Root Mean Square (*RMS*): similar to the *MAV*, the *RMS* feature represents the signal in an average sense. The *RMS* feature is calculated using Equation (10), where $X_{n}$ is the sampling point and *N* is the number of samples in the moving window [60,68].
  (10) $$RMS\;=\;\;\sqrt{\frac{1}{N}\sum_{n\;=\;1}^{N}X_{n}^{2}}\;\;$$
3. Variance (*VAR*): The variance of a signal is related to the deviation of the sampling points from their average, $\bar{x}$ and is calculated by Equation (11). The variance is the mean value of the square of these deviations [60].
  (11) $$VAR\;=\;\;\frac{1}{N-1}\sum_{n\;=\;1}^{N}{\left(x_{n}-\bar{x}\right)}^{2}\;\;$$
4. Waveform length (*WL*): The waveform length is derived by summation over the numerical derivative of the samples and is given by Equation (12) [60,68,69].
  (12) $$WL\;=\;\;\sum_{n\;=\;1}^{N-1}\left|X_{n+1}-X_{n}\right|\;\;\;$$

**Frequency-domain features**

Discrete Fourier Transform (DFT): Since the coordinates were transferred to HCS, it is a valid assumption to assume that the grasps, and therefore the joint coordinates, were constant through an entire task. Therefore, the DFT was used to transfer signals from the time domain to the frequency domain. numpy.fft.fft was used to extract DFT features based on Equation (13), where $W_{N}\;=\;e^{-j2\pi /N}$ [70].

(13) $$\left\{\begin{array}{cc} X\left[k\right]\;=\;\;\sum_{n\;=\;0}^{N-1}\;x\left[n\right]W_{N}^{nk}\;\;\; & k\;\;=\;\;0,\;1,\ldots ,\;N-1\;\\ x\left[n\right]\;=\;\;\frac{1}{N}\sum_{n\;=\;0}^{N-1}\;X\left[k\right]W_{N}^{-nk}\;\;\; & n\;\;=\;\;0,\;1,\ldots ,\;N-1\; \end{array}\right.$$

#### 2.3.2. Classification

The data matrix for each feature was formed by concatenating the features from all trials of all the tasks. The size of the obtained matrix was n×m, where *n* is the number of sampling points from all trials of all tasks and *m* is the number of feature components. Data matrices were standardized to have zero mean and unit variance per column before being fed to the machine learning algorithms.

The SVM is well-known to be a strong classifier for hand gestures [23,44,71,72,73,74,75,76]. It is a robust algorithm for high-dimensional datasets with smaller numbers of sampling points. The SVM maps data into a higher dimensional space and separates classes using an optimal hyperplane. In this study, the scikit-learn library [77] was used to implement the SVM with a Radial Basis Function (RBF), and the parameters were determined heuristically [78].

**Figure 9:**
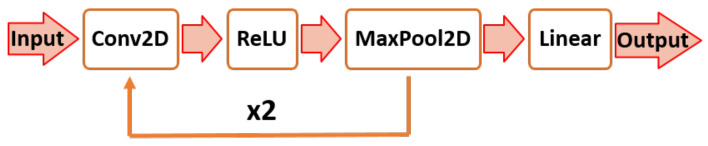
Proposed CNN architecture [54].

Moreover, a Convolutional Neural Network (CNN) was implemented in PyTorch [79,80] for classifying the tasks. CNN and its variations have been shown to be efficient algorithms for hand gesture classification [81,82,83,84]. The proposed architecture of the CNN is illustrated in Figure 9. The CNN architecture is composed of three convolution layers and one linear layer. The three convolution layers have output channels of 16, 32, and 32 in sequential order, and each convolution layer consists of 2 × 2 filters with a stride of 1 and zero padding of 1. The Rectified Linear Unit (ReLU) activation function and batch normalization function were applied at the end of each convolution layer, and the maximum pooling function was applied at the end of the first and second layers. A fifty % dropout was implemented at the end of the fully connected layer, i.e., after the linear function in Figure 9. The learning rate, epoch, and batch size for training the CNN algorithm were set to 0.01, 20, and 40, respectively. The hyperparameters were determined experimentally.

## 3. Results and Discussion

PCA dimensionality reduction, the adaptive learning rate for training the CNN algorithm, and different data filtering schemes were tested and were rejected as they were shown to be detrimental to the classification accuracy. The 5-fold cross validation performance metrics of the CNN and SVM algorithms in classifying the ADL tasks on the pure data, i.e., filtered data in HCS, as well as different combinations of features are presented in Table 3 and Table 4, respectively. The precision, recall, and F1-score were calculated using the sklearn.metrics.precision_recall_fscore_support function by setting average = ‘macro’ to calculate these metrics for each class and report their average values.

Both algorithms were better at classifying some of the time-domain features when compared with their performance when classifying pure data. Among the time-domain, non-geometrical features, VAR and WL represent the data poorly, as they are calculated based on variations in the signal over time (Equations (11) and (12)). Since the data were transformed to HCS, the grasps, and consequently the coordinates of the joints, can be assumed to be constant over time. Therefore, VAR and WL are very similar in different tasks and cannot be used to discriminate tasks from each other. Similarly, DFT features can be assumed to represent the frequency decomposition of DC signals with different amplitudes. As a result, the interclass variability in this feature is not high enough to achieve a high classification accuracy.

Based on Table 3 and Table 4, SVM and CNN have comparable accuracy levels when classifying geometrical features. However, SVM outperforms CNN when features are combined. This could be correlated to the ability of SVM to classify high-dimensional datasets, even when the number of samples is not proportionally high.

The classification accuracies achieved using the AFA and FTE features were lower than those achieved in a similar study [61]; however, the tasks classified in the two studies were very different. The ADL dataset includes many tasks in which the fingers are flexed while the hand holds an object. This minimizes the variation in AFA and FTE among the tasks. In addition, to have a meaningful comparison between the results of different studies, the inclusion or exclusion of gross hand motions in the classification should be taken into account. In the current analysis, information about the gross hand motions was removed from the data.

**Figure 10:**
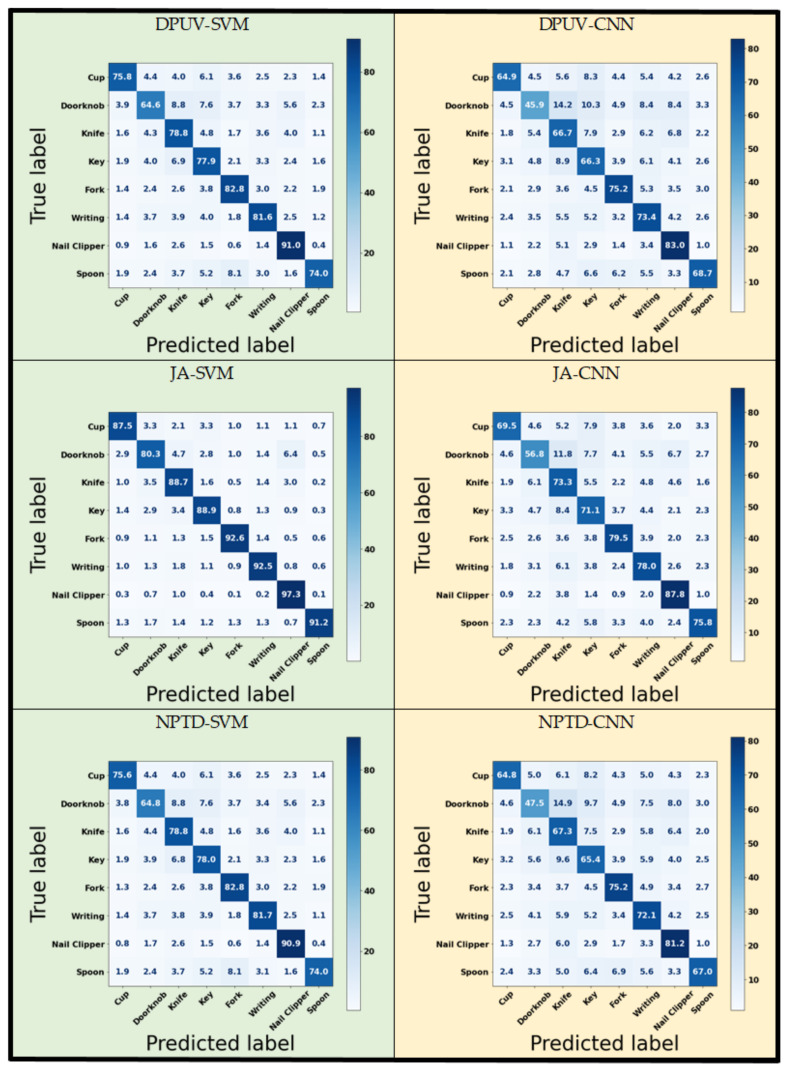
Confusion matrices for sample combinations of features and classifiers. All values were obtained through 5-fold cross validation and are presented as percentages (%).

As demonstrated in Table 3 and Table 4, ATD and JA are the best features for classifying the tasks using both algorithms. The ATD-CNN combination achieved a classification accuracy of over 99% and precision and recall values of over 97%. JA performed better when combined with the SVM algorithm. The JA-SVM combination achieved values of over 90% for both accuracy and precision and a recall of over 89%. Moreover, combining two or more time-domain features can improve the classification performance using the same classifiers. Confusion matrices for both classifiers and sample geometrical features achieved accuracy levels of over 70%, as presented in Figure 10. The uniform distribution of off-diagonal elements in these matrices shows that the algorithms were not overfitted to any of the classes using these features.

## 4. Conclusions and Future Work

In this work, several classification systems were presented. These systems are made from the combination of a variety of time-domain and frequency-domain features with the SVM and CNN used as classifiers. The classification performance of the systems was tested on a proposed ADL dataset. The ADL dataset includes leap motion controller data collected from the upper limbs of healthy adults during the performance of eight common ADL tasks. To the best of authors’ knowledge, this is the first ADL dataset collected by the LMC that includes both static hand grasps and dynamic hand motions of participants using real daily-life objects.

In this work, the data were transformed into HCS, so only the grasp information, and not the gross hand motions, were used for classification. A classification accuracy of over 99% and precision and recall values of over 97% were achieved by applying CNN on the “adjacent fingertips distance” feature. Eleven classification systems achieved a classification accuracy of over 80% with six achieving values of over 90% with high precision and recall values. Although the CNN and SVM had comparable performances for the individual features, for the combination of features, the SVM outperformed the CNN algorithm. From these observations, it can be deduced that the presented CNN algorithm may achieve a greater accuracy level if the size of the ADL dataset is increased.

The findings of this study pave the way for developing an ADL-assessment-metric in two ways. First, these findings can be immediately applied to evaluate a patient’s performance, and secondly, they can have long-term applications.

In the current study, a data analysis pipeline that takes LMC data from hand motions into account and outputs a classification accuracy to distinguish different ADL tasks was developed. Different preprocessing, feature extraction, and classification methods were tested on data collected from healthy adults to detect the best structure and parameters for the proposed pipeline. The developed pipeline can be set as a reference. Then, hand motion data from a neurological patient completing the same tasks with the same data collection setup can be fed into the reference pipeline to obtain the classification accuracy. The achieved accuracy indicates how close a patient’s hand motions are to the hand motions of the healthy population. This method enhances the assessment of the overall performance of a patient in a quantitative fashion. In addition, the acquired confusion matrix provides insight into the patient’s performance when completing each individual task.

As for the long-term applications, the features that achieve higher classification rates can be used for further analysis and for developing other metrics, as they represent different classes in a more distinguishable way. For instance, the distribution of these features in each ADL task among the healthy adults can be set as a reference metric. In this scenario, the location of a patient’s hand data in the reference distribution can be used to evaluate the patient’s performance and the rehabilitation progress. Greater analysis of the data from healthy adults as well as collection of the same data from neurological patients is required to complete this metric.

In conclusion, future work should be focused on three directions. Firstly, other classifiers should be investigated to increase the algorithm’s speed. Furthermore, the LMC data should be transformed back to the global coordinate system to include gross hand motions and implement time series algorithms for classification. Finally, the ADL dataset should be expanded by recruiting more healthy and neurological patients as participants to advance the proposed methodology further toward the development of a quantitative assessment method. Particularly, data from the neurological patients are crucial to generalize the findings of the current study for clinical applications.

## Figures and Tables

**Table 1:** Dynamic tasks of the ADL dataset [54].

Object	Dynamic Task
Cup	Grabbing a cup from the table top and bringing it to mouth to pretend drinking from the cup and put it back on the table
Fork	Bringing pretended food from a paper plate on the table to the person’s mouth
Key	Locking/unlocking a pretended door lock while holding a car key
Knife	Cutting a pretended stake by moving the knife back and forth
Nail Clipper	Holding a nail clipper and pressing/releasing its handles
Pen	Tracing one line of uppercase letter “A”s, with 4 randomly distributed font sizes
Spherical Doorknob *	Rotating a doorknob clockwise and counter clockwise
Spoon	Bringing pretended food from a paper plate on the table to the person’s mouth

* A cup was used instead of a spherical doorknob and the participants were instructed to mimic the hand posture of holding a spherical doorknob.

**Table 2:** Feature categories.

Time-domain	Geometrical	AFA, ATD, DPUV, FHA, FTE, JA, NPTD
	Non-geometrical	MAV, RMS, VAR, WL
Frequency-domain	DFT	
Description of acronyms: Adjacent Fingertips Angle(AFA), Adjacent Tips Distance (ATD), Distal Phalanges Unit Vectors (DPUV), Fingertip-$\vec{h}$ Angle (FHA), Fingertip Elevation (FTE), Joint Angle (JA), Normalized Palm-Tip Distance (NPTD), Mean Absolute Value (MAV), Root Mean Square (RMS), Variance (VAR), Waveform Length (WL), Discrete Fourier Transform (DFT)		

**Table 3:** Performance metrics for different combinations of features with the CNN as a classifier using 5-fold cross validation. All numbers are presented as percentage values (%). Different sets of features, based on Table 2, are shown in different colors.

Feature	CNN			
	Accuracy	Precision	Recall	F-Score
**Pure data**	63.5	50.5	40.2	41.2
**MAV**	85.1	80.5	80.1	80.2
**RMS**	84.1	78.1	77.8	77.9
**VAR**	34.8	32.9	23.5	23.3
**WL**	36.7	31.4	29.2	29.6
**AFA**	57.3	54.4	52.3	52.6
**ATD**	99.88	97.5	97.3	97.4
**DPUV**	72	68.8	68	68.3
**FHA**	70.2	66.1	65.3	65.5
**FTE**	41.5	29	25.4	24.5
**JA**	77.4	74.4	73.9	74.2
**NPTD**	71.5	68.4	67.6	67.9
**DFT**	58.4	53.4	50.4	51.4
**JA+DPUV**	80.4	77.1	76.6	76.8
**JA+NPTD**	78.8	74.3	73.7	74
**MAV+RMS**	84	79.5	78.9	79.2
**MAV+JA+NPTD**	88.4	83.8	83.6	83.7
**MAV+JA+NPTD+DPUV**	87.59	82.9	82.5	82.7

**Table 4:** Performance metrics for different combinations of features with the SVM as a classifier using 5-fold cross validation. All numbers are presented as percentage values (%). Different sets of features, based on Table 2, are shown in different colors.

Feature	SVM			
	Accuracy	Precision	Recall	F-Score
**Pure data**	68.9	70.5	67.3	68.2
**MAV**	79.5	81.1	77.9	78.99
**RMS**	76.3	78.4	74.7	75.8
**VAR**	24.6	61.1	21.8	20.3
**WL**	29.4	48.7	26.7	25.6
**AFA**	49.6	57.1	46.9	47.5
**ATD**	75.1	80.3	74.2	76.1
**DPUV**	79.3	79.2	78.3	78.7
**FHA**	64.2	66.2	62.5	63.3
**FTE**	30.8	50.8	29	30.4
**JA**	90.3	90.2	89.9	90
**NPTD**	79.3	79.2	78.3	78.6
**DFT**	52.4	77.6	50	54.3
**JA+DPUV**	94.7	94.4	94.4	94.4
**JA+NPTD**	92.3	92.2	91.9	92
**MAV+RMS**	79	80.5	77.5	78.4
**MAV+JA+NPTD**	92.5	92.3	91.9	92
**MAV+JA+NPTD+DPUV**	95.1	94.8	94.7	94.8
